# A Computationally Lightweight Algorithm for Deriving Reliable Metabolite Panel Measurements from 1D ^1^H NMR

**DOI:** 10.1021/acs.analchem.1c00113

**Published:** 2021-03-18

**Authors:** Panteleimon G. Takis, Beatriz Jiménez, Nada M. S. Al-Saffar, Nikita Harvey, Elena Chekmeneva, Shivani Misra, Matthew R. Lewis

**Affiliations:** † National Phenome Centre, Imperial College London, Hammer-smith Campus, IRDB Building, London W12 0NN, United Kingdom; ‡ Section of Bioanalytical Chemistry, Division of Systems Medicine, Department of Metabolism, Digestion and Reproduction, Imperial College London, South Kensington Campus, London SW7 2AZ, United Kingdom; # Section of Metabolic Medicine, Division of Diabetes, Endocrinology and Metabolism, Department of Metabolism, Digestion and Reproduction, Imperial College London, St. Mary’s Campus, London W1 1PG, United Kingdom

## Abstract

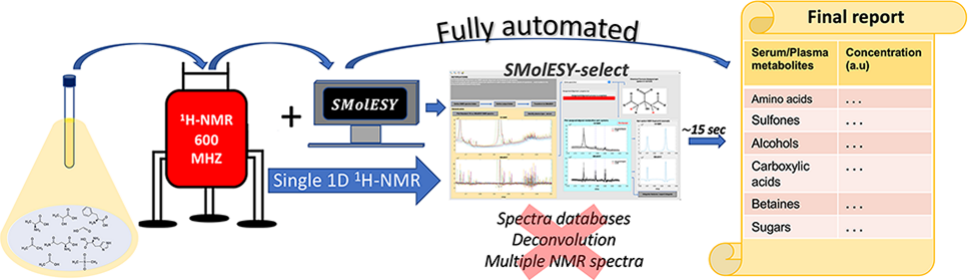

Small Molecule Enhancement SpectroscopY (SMolESY) was employed to develop a unique and fully automated computational solution for the assignment and integration of ^1^H nuclear magnetic resonance (NMR) signals from metabolites in challenging matrices containing macromolecules (herein blood products). Sensitive and reliable quantitation is provided by instant signal deconvolution and straightforward integration bolstered by spectral resolution enhancement and macromolecular signal suppression. The approach is highly efficient, requiring only standard one-dimensional ^1^H NMR spectra and avoiding the need for sample preprocessing, complex deconvolution, and spectral baseline fitting. The performance of the algorithm, developed using >4000 NMR serum and plasma spectra, was evaluated using an additional >8800 spectra, yielding an assignment accuracy greater than 99.5% for all 22 metabolites targeted. Further validation of its quantitation capabilities illustrated a reliable performance among challenging phenotypes. The simplicity and complete automation of the approach support the application of NMR-based metabolite panel measurements in clinical and population screening applications.

Metabolic profiling technologies are powerful in their ability to represent the chemical complexity of human biofluids and tissue extracts with both analytical specificity and breadth.^1^ Proton nuclear magnetic resonance (^1^H NMR) spectroscopy is one of the principal analytical tools used for metabolic profiling of biofluids owing to its minimal requirement for sample preparation, ease of automation, accurate quantitation, robustness, and reliability.^2^ Together, these qualities make NMR ideal for both population screening and translation into clinical environments for use in patient health monitoring and diagnostics.^3^ However, the interpretation of ^1^H NMR profiles is often brokered by dedicated bioinformatic data processing efforts required to reduce data complexity (e.g., bucketing) as well as align, normalize, and assign chemical identity to the signals (resonances) detected. These steps often disconnect and distance powerful metabolic measurement data from the clinicians, dietitians, biologists, epidemiologists, etc. who could otherwise more directly interact with the data to investigate their hypotheses. Therefore, to propel the technique’s utility and application in these research areas, automated solutions are needed to reliably elucidate NMR profiling data in a readily interpretable form as metabolite panel measurements.

To achieve this, the causes of variation in the underlying NMR profile, which hinder automated metabolite quantitation, must be considered. The sensitivity of NMR profiles to the chemical composition of each sample potentially complicate the prerequisite chemical annotation of resonances due to chemical shift (δ) variations and resolution.^4,5^ Fortunately, the homeostatic control of blood, in contrast to urine, ensures a relatively stable chemical composition, and δ variation is further controlled in blood product analysis by the addition of a common buffer to the sample.^6^ However, the macromolecular content (e.g., lipids and proteins) of blood product samples yields broad signals that overlap and hinder the facile assignment of signals from small molecule (SM) species.^7^ Additionally, abundant macromolecules facilitate the exchange between bound and free SMs, which broadens line widths (Δ*v*_1/2_) and degrades spectral resolution, further complicating the automated assignment and integration of SM-derived ^1^H NMR signals.^8^

Overcoming these obstacles has conventionally required additional experiments beyond the standard 1D ^1^H NMR including a spin–echo experiment (e.g., Carr–Purcell–Meiboom–Gill, CPMG, pulse sequence^9^) for the attenuation of macromolecular signals and a pseudo 2D (e.g., Jres). The combination of these NMR experiments comprising the now-standard metabolomics pipeline, when augmented by additional 2D validation experiments, has been sufficient to support the assignment of >36 metabolites in serum/plasma samples.^10^ More recently, strong magnetic field analysis was applied to concentrated and pooled samples after physical removal of the macromolecules, expanding the number of assigned metabolites to 67^8^ and pushing the boundaries of what is detectable by NMR at the expense of routine applicability. Solutions for more routine targeted assignment and quantitation of selected SMs have emerged, including the commercial Bruker IVDr algorithm^7^ which employs both 1D- and 2D-Jres spectra for the automated assignment/quantification of up to 40 plasma/serum metabolites. Other commercial or freely available software require either extensive sample preprocessing^11^ (i.e., manual protein/lipids removal) or the construction of high-quality reference spectra databases, which require manual intervention for Δ*v*_1/2_ or baseline fitting adjustments to ensure applicability to real world samples.^12,13^

Recently, we introduced Small Molecule Enhancement SpectroscopY (SMolESY),^5^ a highly validated computational approach that exclusively utilizes the standard 1D ^1^H NMR experiment to derive enhanced SM profiles from macromolecule-rich sample types such as serum and plasma. SMolESY maintains both the qualitative and quantitative features of the original experiment while suppressing the macromolecular signal and increasing spectral resolution (see more details in the Supporting Information). These enhancements benefit the interpretation of the SM profile, demonstrated here among amino acids and sugars with ^1^H NMR signals otherwise invisible or barely visible in the CPMG profile (Figure 1a–c).

**Figure 1:**
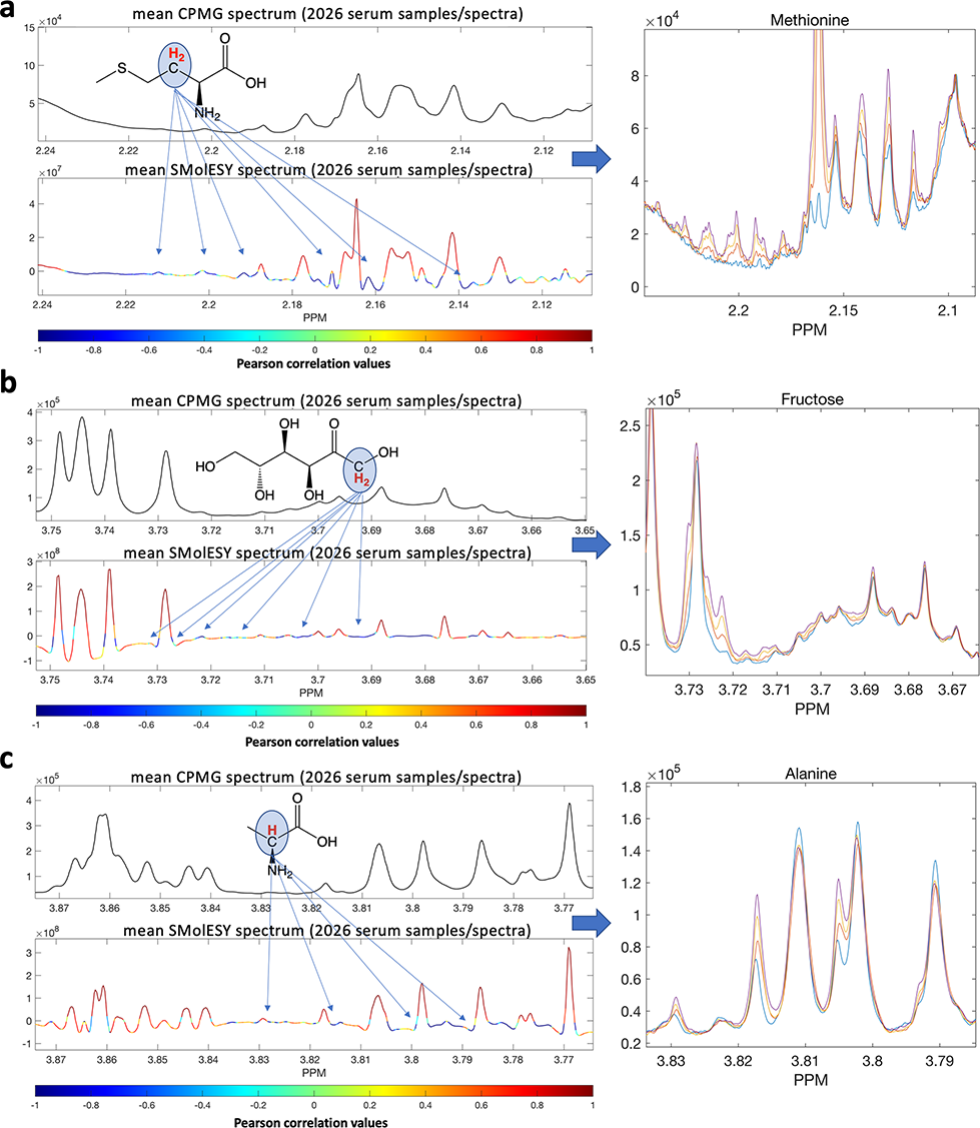
Pearson linear correlation between 2026 plasma CPMG and SMolESY ^1^H NMR spectra. Correlation between each SMolESY feature versus its corresponding one in the CPMG shows the enhanced SM signals, highlighting the detection ability of SMolESY with respect to the spin–echo experiment (e.g., CPMG). Low correlated features correspond to being barely visible/highly overlapped by other signals/baseline in the CPMG, whereas in SMolESY, they are partially or totally deconvolved. By employing 2D ^1^H NMR correlation spectroscopy (COSY) and statistical correlation spectroscopy, (STOCSY;^15^Figure S1), we were able to assign several of these features, which were subsequently confirmed by spiking experiments (left-hand panels). Examples of resonances now visible in the SMolESY spectrum are shown in the right-hand panels: (a) methionine, (b) fructose, and (c) alanine.

Building on these observations, we report a novel, automated, and reliable approach to metabolite panel measurement in human plasma/serum using only the standard 1D ^1^H NMR experiment and SMolESY. The algorithm (SMolESY-select) has been validated for the assignment and integration of resonances from one or more ^1^H NMR spin systems across 22 clinically important metabolites (Table S1) in plasma/serum profiles. It accomplishes this without requiring the construction and/or adaptation of databases, additional NMR experiments, and any need for complex and computationally expensive deconvolution algorithms. Consequently, SMolESY-select delivers readily interpretable relative serum/plasma metabolite quantification with significantly reduced time and cost compared to alternative approaches. The freely available algorithm is suitable for 1D ^1^H NMR data acquired using widely established NMR sample preparation and spectra acquisition protocols/SOPs (see the experimental details in the Supporting Information).

## Experimental Section

Reagents for NMR sample preparation were purchased from Sigma-Aldrich, and NMR samples were acquired using Bruker IVDr 600 MHz spectrometers. Further details as well as computational/functional features of SMolESY-select are reported in the Supporting Information.

## Results and Discussion

**Figure 2:**
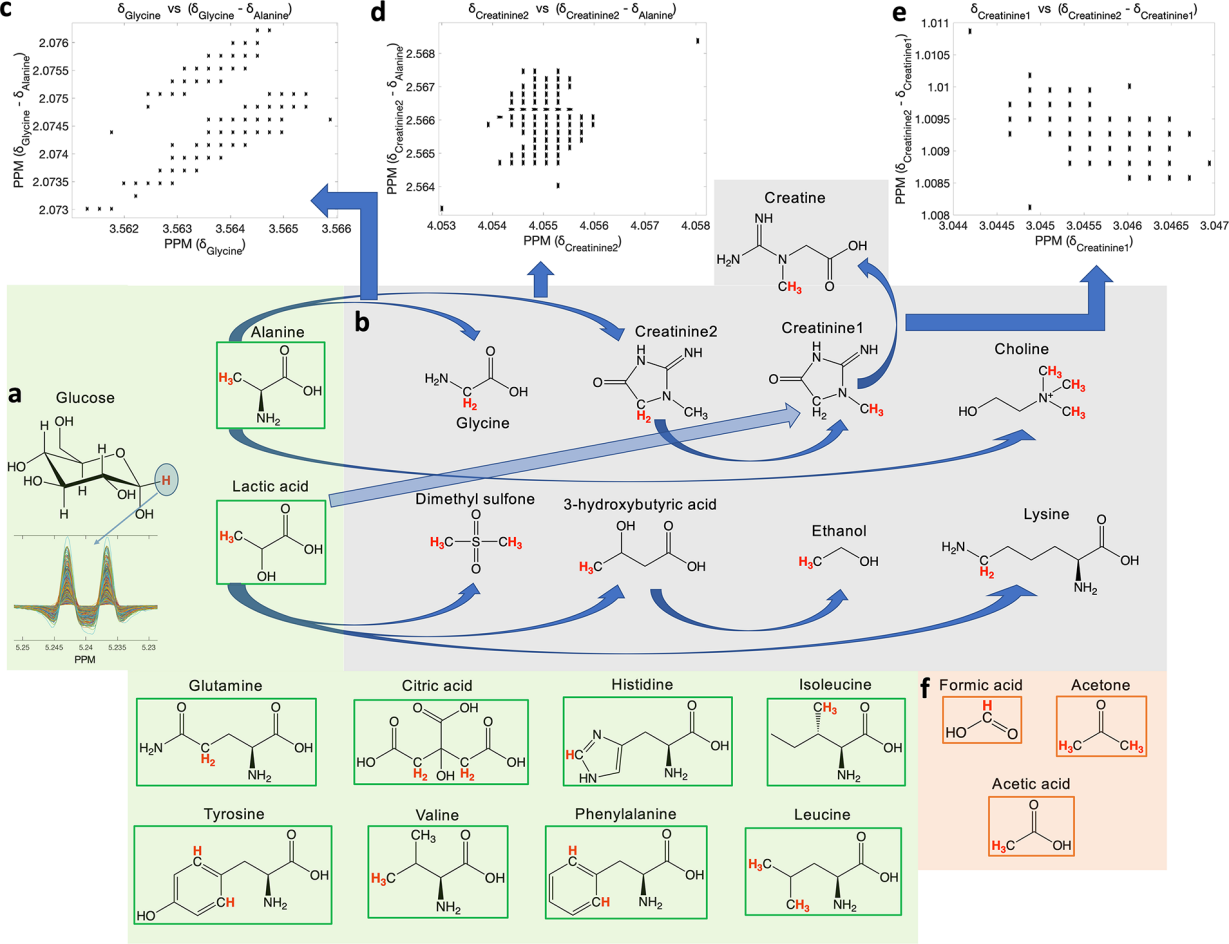
Employed strategy for the automated assignment of 22 serum/plasma metabolites. (a) ^1^H NMR/SMolESY spectra are calibrated to the glucose anomeric proton doublet. Signals corresponding to the ^1^H highlighted in red font are used for assignment/quantitation of all metabolites. The glucose doublet and metabolites in the green boxes are assigned by pattern recognition (e.g., by imposing *J*-coupling constraints) in previously defined spectral windows with a width ≥0.01 ppm at 600 MHz. (b) Simple correlations, based upon 4023 plasma/serum unique spectra, with alanine and lactic acid methyl group signals are used as assignment constraints for metabolites in gray squares, which cannot otherwise be assigned using *J*-coupling constraints because they present either singlets or multiplets whose SMolESY components have a high risk of overlap (Table S2 and Figure S2). (c) Glycine singlet assignment is further supported by the minimization of the predefined spectral window owing to the decrease in line broadening achieved via SMolESY (≤0.004 ppm at 600 MHz). (d) Assignment of creatinine requires all previous constraints plus (e) extra correlations between intra molecular ^1^H NMR spin systems (e.g., between the −CH_3_ and −CH_2_ groups of creatinine). (f) The singlets from acetic acid, acetone, and formic acid were not found to significantly correlate with any other abundant metabolite; however, the predefined windows for these metabolites’ signals were sufficiently narrow (≤0.006 ppm for acetone/acetate and ≤0.008 ppm for formic acid) following spectral calibration to glucose which, combined with SMolESY and the general homeostatic nature of blood matrices, allows for their reliable identification.

Figure 2 summarizes the steps followed for metabolite assignment. Initially, all spectra are automatically calibrated to the ^1^H NMR signals of glucose, in particular, to the anomeric proton of glucose (Figure 2a) resonating at 5.233 ppm as routinely done for the analysis of blood ^1^H NMR profiles.^14^ Next, the algorithm searches the SMolESY data for each spin system pattern within defined spectral windows (bins). Bins were defined for each of the 24 ^1^H NMR spin systems using a cohort of 4023 unique plasma/serum samples (3023 plasma and 1000 serum) and employing several statistically-based tools (e.g., STOCSY, Figure S1), spiking experiments, and additional multidimensional NMR experiments (e.g., Jres) to validate each NMR signal assignment per spectrum. In routine operation, the assignment process is straightforward for multiplets (i.e., doublets, triplets, etc.) (Figure 2a) owing to both applied *J*-coupling constraint and SMolESY resolution enhancement. The automatic identification of the ^1^H NMR singlets is more challenging, including those belonging to particular chemical groups of glycine, creatine/creatinine, choline, dimethyl-sulfone (DMSO2), acetone, and acetic acid (Figure 2b). The width of the selected bins encompassing the NMR signals of these spin systems consisted of at least 10 SMolESY Δ*v*_1/2_ (>0.01 ppm, where 0.0009 < SMolESY Δ*v*_1/2_ < 0.0011 ppm for the 600 MHz NMR instrument), resulting in a high risk of misassignment. To mitigate this, a strategy similar to that published previously for the δ prediction of urine metabolites^4^ was pursued whereby the δ values of singlets were correlated with those from the most abundant and frequently occurrent serum/plasma metabolites.^10^ Specifically, singlets from glycine, creatinine, choline, and DMSO2 were correlated among the 4023 serum/plasma spectra with signals from lactic acid and alanine (Figure 2b). The approach yields significantly smaller spectral bins for the above-mentioned metabolites with a maximum width of 3–5 SMolESY Δ*v*_1/2_ (Figure 2c,d), facilitating the assignment of metabolite signals including those from creatine and ethanol (Figure 2b,e). The spectral window ranges for acetone/acetate and formic acid NMR signals (Figure 2f) were already narrow enough (i.e., <5 and <7 SMolESY Δ*v*_1/2_, respectively), which combined with the homeostatic nature of blood products, minimize the risk of misassignment for the corresponding metabolite signals. Further details of various NMR signal automated assignment are described in Table S2 and Figure S2.

**Figure 3:**
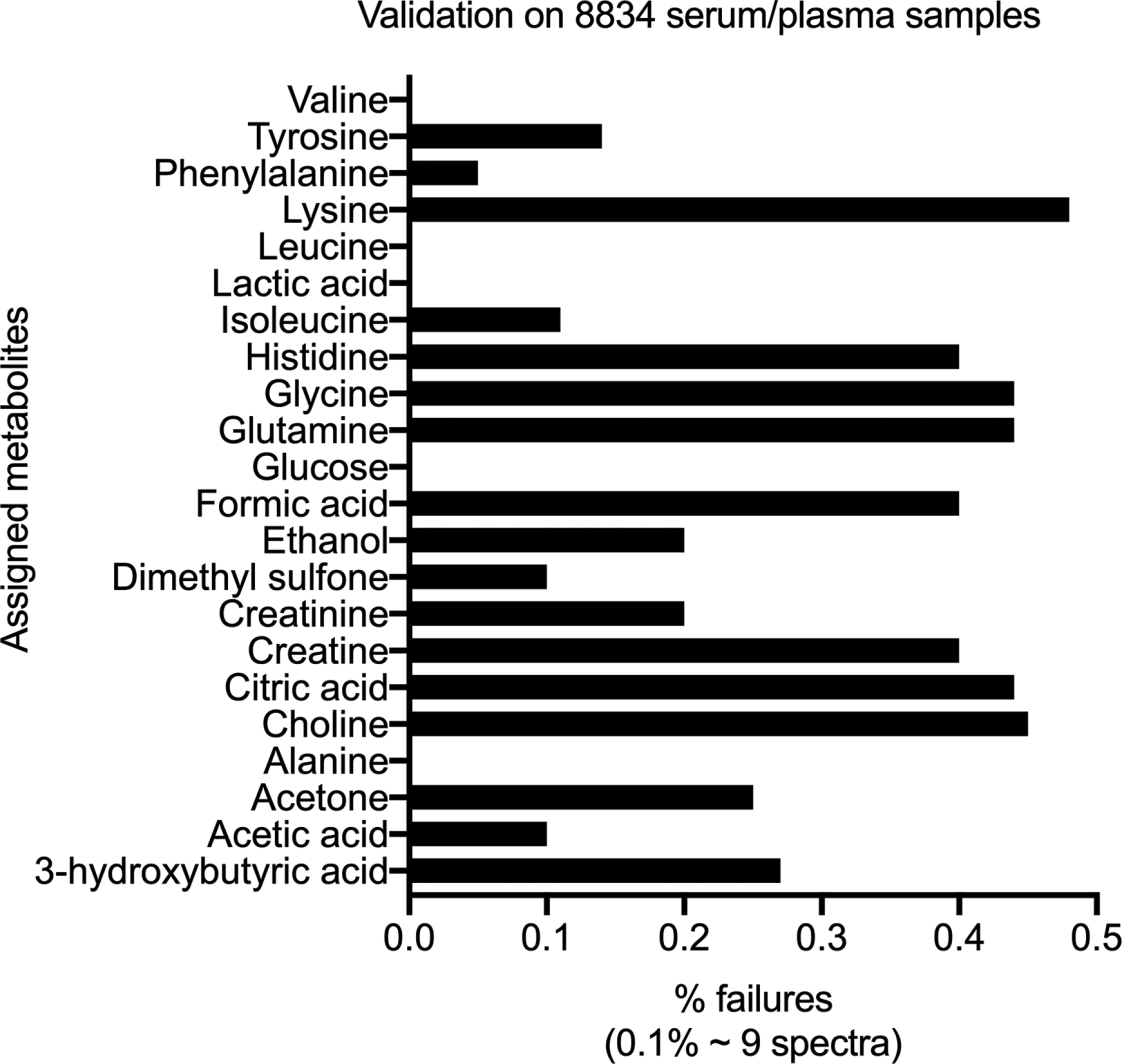
Performance of the automated assignment applied to 8834 serum/plasma samples, illustrated as a percentage of failures (i.e., wrong and/or missed assignments of SMolESY features) for each metabolite.

A smoothing filter is then applied within selected NMR spectral windows to help with the recovery of signals belonging to frequently low abundance metabolites, which appear in noisy spectral regions (i.e., aromatic region, around the suppressed H_2_O signal, etc.) (Figure S3a).^5^ Specifically, SMolESY-select utilizes a moving average filter spanning 11-points (see the Supporting Information) across the high resolution data of the selected windows, which increases the s/n of signals belonging to tyrosine, phenylalanine, histidine, creatinine, and choline (Figure S3b),^16^ while maintaining the attenuation of macromolecular signals/baseline background at the slight expense of resolution enhancement. The denoised SMolESY signals still maintain a slightly narrower Δ*v*_1/2_ compared to the standard 1D ^1^H NMR experiment, and their s/n is a maximum of ∼10% less than that of the CPMG spectra (Figure S3b,c). The metabolite assignment capability of the SMolESY-select algorithm was extensively validated using an independent cohort of 8834 blood product samples consisting of 5338 plasma and 3496 serum spectra from our internal databases (see the experimental details in the Supporting Information) as well as one serum cohort from the Metabolights repository (https://www.ebi.ac.uk/metabolights) (MTBLS395). Several examples of the assigned 24 ^1^H NMR spin systems’ signals from 22 metabolites are reported in Figure S4a–j. Note that SMolESY-select aligns only each assigned spin system to a random ppm value within their corresponding predefined spectral window, easing the visual detection of any misassignment. For the 22 metabolites in the unique 8834 blood serum/plasma spectra, false positive or nonassigned NMR signals ranged from zero to a maximum of 40 (i.e., 0–0.45% failure) (Figure 3), highlighting the accuracy and robustness of the method. It is noteworthy that SMolESY succeeded in recovering barely visible signals of metabolites in the standard 1D ^1^H NMR experiment (Figure S4h), increasing indirectly the limit of detection for specific ^1^H NMR spin systems’ signals.

The use of SMolESY, with its baseline suppression and resolution enhancement features, enables the direct integration of the assigned spectral components, precluding the need for complex signal deconvolution or baseline fitting, substantially improving the efficiency of computation. To achieve this, a thorough examination of the 4023 plasma/serum 1D spectra was conducted to evaluate the overlap of each spin system’s SMolESY pattern with other SM signals using STOCSY and 2D-Jres analyses for the 22 metabolites (Figure S1b,c) when needed. Only the most reliable components of the SMolESY features from each metabolite were selected, defining the final set used by the algorithm for automated integration (Figure S5).

**Figure 4:**
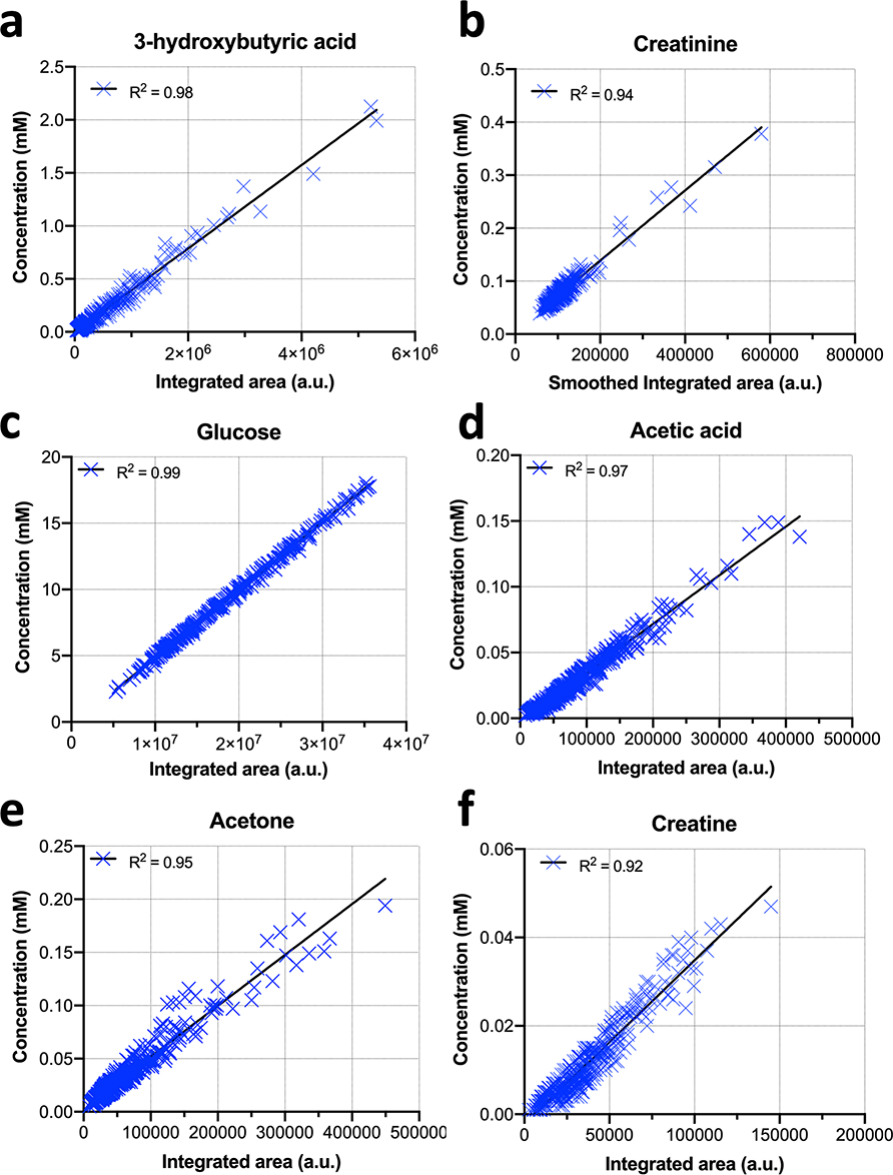
Linear regression analyses of SMolESY integrals (normalized to one proton) versus absolute concentrations produced by commercial software for (a–f) six metabolites in 380 plasma samples from a diabetic cohort. The calculated *R*^2^ values indicate a good correlation between SMolESY feature integrals (independently of smoothing, e.g., panel (b)) and the concentration values from “expensive” fitting/deconvolution processes. Additional comparisons are presented in Figure S7.

The initial validation of the method’s performance was performed by standard addition of the 22 metabolites to a single representative plasma sample, yielding exceptional correlation (*R*^2^ > 0.98) with automatically calculated integrals (Table S3, Figure S6). To further validate integration performance in the context of real-world application, SMolESY-select was applied to a selected subset (see the experimental details in the Supporting Information) of ∼380 plasma/serum spectra from a diabetic cohort, specifically challenging the spectral deconvolution due to abundant glucose and low abundance of selected metabolites.^17^ Results were compared with those obtained from absolute concentration values provided by successful and manually validated application of the established commercial standard approach, Bruker IVDr (Figures 4 and S7),^7^ which requires all three routine experiments (1D ^1^H NMR, CPMG, and Jres) and polynomial fitting for baseline removal. Linear regression revealed a good correlation for several tested metabolite integrals (0.89 < *R*^2^ < 0.99) (Figures 4 and S7a–k). Additionally, SMolESY-select integrals were compared with independent, biochemically measured concentrations using routine clinical methods yielding a linear correlation (Figure S7l,m). When compared with a biomarker discovery approach utilizing the whole ^1^H NMR profile, principal component analysis (PCA) of the 22 plasma metabolites’ relative concentration values from SMolESY-select in a cohort of 361 plasma samples consisting of two independent NMR data sets efficiently reveals the same biomarkers (Figure S8). Together, these analyses demonstrate the direct applicability and reliability of the algorithm present for the 22 metabolites’ panel measurements in the serum/plasma matrices.

## Conclusions

In summary, we introduced a unique algorithm, SMolESY-select, which demonstrates exclusive advantages and significantly contributes to the NMR-based metabolomics pipeline by the sizable decrease of both experimental and computational time required to generate reliable metabolite panel measurements. Here, the relative concentrations of 22 serum/plasma metabolites are efficiently produced from human serum/plasma samples without computationally expensive algorithms, database construction, laborious sample preprocessing (i.e., protein removal), or manual intervention. The analysis is fully automated and requires, on average, less than 15 s per spectrum on a conventional laptop, dramatically outperforming fitting and deconvolution approaches that require several minutes of computation per spectral set. It leverages the performance characteristics of the SMolESY application to standard 1D ^1^H NMR spectra without further dependency on additional NMR data types. This in turn increases the potential throughput of NMR metabolite panel measurement and maximizes the utility of existing 1D ^1^H NMR data sets without preventing the traditional discovery analysis approach^2^ of either the regularly processed or SMolESY enhanced profiles.^5^ These advantages are particularly important in epidemiology, biobanking research applications that require the sequential automated analysis of thousands of human samples and the translation of NMR into clinical practice. The performance of SMolESY-select has been extensively validated across >8800 serum/plasma samples of varying phenotypes. Its use is supported by a graphical user interface (GUI) (Figure S9, Video S1) requiring minimal prerequisite knowledge to operate, and it is freely available to download at https://github.com/pantakis/SMolESY-select.

## Supporting Information Available

SMolESY details; NMR experiments/apparatus; reagents; NMR sample preparation SOPs; computational details software; ethics declarations for the serum/plasma cohort studies; selected metabolites (Table S1); STOCSY examples (Figure S1); autoassignment details (Figure S2, Table S2); smoothing filter details (Figure S3); examples of several assigned ^1^H NMR spin systems (Figure S4); SMolESY patterns and components for integration (Figure S5); validation of SMolESY-select quantitative ability: against experimental data (Figure S6, Table S3); validation of SMolESY-select quantitative ability: against quantitation results by commercial software and clinical methods (Figure S7); combination of targeted/untargeted multivariate analysis on NMR data sets (Figure S8); overview of the SMolESY-select graphical user interface (GUI) (Figure S9) (PDF)
Video S1: SMolESY-select demonstration/functionalities (MP4)
