# Supplementary material for: A Computationally Lightweight Algorithm for Deriving Reliable Metabolite Panel Measurements from 1D 1H NMR
Source: Anal Chem. 2021 Mar 18;93(12):4995–5000. doi: 10.1021/acs.analchem.1c00113 (PMC8041249; doi:10.1021/acs.analchem.1c00113)
Supplement: Supplementary file 1 — ac1c00113_si_001.pdf [file ac1c00113_si_001.pdf]

SUPPORTING INFORMATION

A computationally lightweight algorithm for deriving reliable metabolite panel measurements from 1D ^{1}H NMR

Panteleimon G. Takis,[†,‡,*] Beatriz Jiménez,[†,‡] Nada M. S. Al-Saffar,[†,‡] Nikita Harvey,[†,‡] Elena Chekmeneva,[†,‡] Shivani Misra,[#] and Matthew R. Lewis[†,‡]

[†]National Phenome Centre, Imperial College London, Hammer-smith Campus, IRDB Building, London, W12 0NN, UK

[‡] Section of Bioanalytical Chemistry, Division of Systems Med-icine, Department of Metabolism, Digestion and Reproduction, Imperial College London, South Kensington Campus, London, SW7 2AZ, UK

[#]Section of Metabolic Medicine, Division of Diabetes, Endocrinology and Metabolism, Department of Metabolism, Digestion and Reproduction, Imperial College London, St. Mary's Campus, London, W1 1PG, UK

[*]Correspondence and any requests for materials should be addressed to Dr. Panteleimon G. Takis,

e-mail: p.takis@imperial.ac.uk

Table of Contents Pages

Small Molecule Enhancement SpectroscopY ***S3***

Experimental-computational information ***S4***

Reagents *S4*

NMR sample preparation *S4*

NMR Profiling acquisition details *S4*

2D COSY ^1H NMR spectra acquisition details *S4*

NMR spectra quality control *S4*

Computational details - Software *S4*

Selection criteria for the subset of spectra from the diabetic cohort *S4*

Glucose and creatinine measured concentrations by biochemical methods *S5*

Ethics declaration – plasma/serum cohort studies employed for SMolESY-select *S5*

Selected serum/plasma metabolites (n = 22) ***S6***

Table S1. *The current panel of metabolites provided by SMolESY-select software – examples of their clinical-metabolic importance* *S6*

STOCSY examples for metabolites assignment and signals overlap detection on SMolESY profiles ***S8***

Figure S1. *Examples of several metabolites assignment via STOSCY applied on 4023 plasma/serum SMolESY spectra.* *S8*

Further details for automated ^1H NMR signals assignment ***S9***

Table S2. *The chemical shifts of ^1H NMR spin systems from specific metabolites and their correlation on other spin systems chemical shifts.* *S9*

Figure S2. *Examples of 5 metabolites singlets assignment based upon their correlation with doublets of lactic acid and alanine methyl groups.* *S9*

SMolESY smoothing filter ***S10***

Figure S3. *Examples of less populated plasma/serum NMR spectral regions with small molecule sharp NMR signals* *S10*

Small Molecule Assignment Validation and Spiking Experiments ***S11***

Figure S4. *Examples of several assigned ^1H NMR spin systems for various metabolites in more than one hundred spectra and assignment validations by spiking experiments* *S11*

SMolESY patterns and components for integration ***S13***

Figure S5. *Selected SMolESY components used for integration (i.e. relative quantification) for each ^1H NMR spin system SMolESY pattern* *S13*

Validation of SMolESY-select Quantitative Ability: Against experimental Data ***S14***

Figure S6. *Linear regression analyses between the integrals/proton of the 22 serum/plasma metabolites that are automatically assigned and relatively quantified* *S14*

Table S3. *The seven spiked concentration values (for the case of alanine nine spiked concentrations were used) for each metabolite* *S15*

Validation of SMolESY-select quantitative ability vs. automated quantification by commercial software and clinical measurements ***S16***

Figure S7. *Linear regression analyses of calculated integrals of SMolESY features (normalised to one proton) by our software versus the absolute concentration values calculated by commercial software via fitting/deconvolution algorithms* *S16*

Combination of targeted/untargeted multivariate analysis on NMR datasets ***S17***

Figure S8. *Scores and loadings plots from the PCA analysis of a plasma cohort consisted of two independent NMR plasma-EDTA datasets.* *S17*

Overview of the SMolESY-select graphical user interface (GUI) ***S18***

Figure S9. *Overview of SMolESY-select graphical user interface (GUI)* *S18*

References ***S20***

Small Molecule Enhancement SpectroscopY

Small Molecule Enhancement SpectroscopY (SMolESY),[1] was recently introduced as a novel application of computationally suppressing NMR signals of macromolecules, which functionally supplants the traditional spin-echo NMR experiments by a computational transformation of routinely generated ^{1}H NMR (e.g. 1D-NOESY) dispersion data (i.e. the Fourier transformed imaginary spectral part). SMolESY is based upon traditional mathematical differentials (application of the 1st derivative on the dispersion spectral data, providing complete suppression of the macromolecular signals and enhanced spectral resolution while retaining the quantitative nature of the original ^{1}H NMR (e.g. 1D-NOESY) data. The resulting spectra with cleaner baseline support a reliable signal integration (precluding the need for complex and computationally demanding algorithms for the majority of the metabolite signals) which expedites the chemical assignment and quantification of several metabolites. More importantly, the application of SMolESY on thousands of plasma/serum/food extracts/macromolecular mixtures etc. showed no loss of metabolic information at least in the commonly employed biological matrices with highly abundant macromolecules (e.g. blood plasma/serum), exhibiting the required reproducibility for metabolomics and any analytical studies, greatly improving the quantitative (qNMR) and qualitative NMR analytical assets, while dramatically reducing the instrument time and resource required. SMolESY data could be produced and further explored by a freely available software, allowing its fast implementation on NMR-based metabolomics/analytical studies: https://github.com/pantakis/SMolESY_platform.

Experimental-computational information

Reagents

All reagents used for spiking experiments and NMR sample preparation (e.g. for buffer composition etc.) were purchased from Sigma-Aldrich and are of high purity.

NMR sample preparation

The total number of plasma/serum as well as the spiked (~9000 samples) NMR samples were prepared following the established standard operating procedures for metabolomics analyses.[2] In detail, the plasma NMR samples consisted of 50% plasma buffer [75 mM Na_2HPO_4; 6.2 mM NaN_3; 4.6 mM sodium trimethylsilyl [2,2,3,3-d4]propionate (TMSP) in H_2O with 20% (v/v) ^2H_2O; pH 7.4] and 50% of blood serum/plasma.

NMR Profiling acquisition details

Solution ^1H NMR spectra of all samples were acquired using a Bruker IVDr 600 MHz spectrometer (Bruker BioSpin) operating at 14.1 T and equipped with a 5 mm BBI probe with ^2H decoupling probe including a z-axis gradient coil, an automatic tuning-matching (ATM), high level shimming and an automatic refrigerated sample handling robot (Sample-Jet). Temperature was regulated to 310 ± 0.1 K.

For each blood sample, three NMR experiments were acquired in automation: a general profile ^1H NMR water presaturation experiment using a one-dimensional pulse sequence where the mixing time of the 1D-NOESY experiment is used to introduce a second presaturation time, a spin echo edited experiment using the Carr–Purcell–Meiboom–Gill (CPMG) pulse sequence which filters out signals from fast T_2 relaxing protons from molecules with slow rotational correlation times such as proteins and other macromolecules, and a 2D *J*-resolved experiment (2D-JRES). Each experiment had a total acquisition time of approximately four minutes [32 scans were acquired for the 1D-NOESY (98304 data points, spectral width of 18029 Hz) and the 1D-CPMG (73728 data points, spectral width of 12019 Hz) experiments while two scans and 40 planes were acquired for the 2D J-RES experiment]. Relaxation time was set to 4 s on the 1Ds and 2 s for the pseudo-2D. Spectral width was always set onto the water resonance and optimised for every dataset in order to optimise water signal depletion.

2D COSY ^1H NMR spectra acquisition details

^1H-^1H COSY's were acquired in the above-described spectrometer. Relaxation delay was reduced to 2 s to decrease the experimental time and the pulse sequence included a presaturation period to suppress the water signal with the centre of the window being set to the H_2O chemical shift of the solvent. The spectral window was set to 12.0 ppm with 4096 in the direct dimension, 256 planes and 128 scans.

NMR spectra quality control

All employed 1D ^1H NMR serum/plasma spectra were of high quality and resolution (> 65k data points) which is the established norm within modern high quality metabolomics and analytical studies and were quality controlled using the *nPYc* toolbox.[3]

Computational details - Software

All scripts for spectrum analyses were coded in the MATLAB programming suite (MathWorks, version R2019b). The linear regression analyses as well as their plotting, were performed by Prism 8 (GraphPad Software, Inc, 2019).

Selection criteria for the subset of spectra from the diabetic cohort

The employed 380 spectra for the comparison of SMolESY-select automated integration results with the absolute concentrations of the 22 metabolites measured by Bruker IVDr software (B.I.Quant-PS 2.0™) were selected based upon a thorough inspection of the fitting results provided by the commercial software, where it was confirmed that metabolites signals are correctly assigned and properly fitted. In addition, it was ensured that for the majority of the selected samples biochemically measured concentration values are provided for several metabolites (i.e. creatinine, glucose).

Glucose and creatinine measured concentrations by biochemical methods

Glucose and creatinine were independently measured on research participants with diabetes in ~40 different hospital biochemistry laboratories as part of the MY DIABETES study. All hospital biochemistry labs were UKAS accredited and used routine glucose and creatinine assays suitable for patient standard measurement, calibrated to relevant International Federation of Clinical Chemistry (IFCC) standards and aligned to reference methods and materials, with adequate internal quality control and external quality assurance.

Ethics declaration – plasma/serum cohort studies employed for SMolESY-select

Plasma and/or serum samples spectra employed for the building/validation of SMolESY-select software were selected from National-Imperial Phenome centre internal databases acquired for the following project studies (including ethical statements):

1) MY DIABETES study
   Analysis was undertaken as part of an Imperial Biomedical Research Council, ITMAT funded project (Improving stratification of young-onset diabetes: a multimodal, precision-based approach) using samples from the MY DIABETES study (MODY in Young-Onset Diabetes in Different Ethnicities), clinical trial no. NCT02082132, ClinicalTrials.gov, ethics approval from Chelsea LREC 13/LO/0944 Sep 2013. The MY DIABETES study was funded through a Sutherland-Earl Fellowship from the Diabetes Research & Wellness Foundation.
   This article includes independent research supported by the NIHR BRC at Imperial College Healthcare NHS Trust. The views expressed are those of the author(s) and not necessarily those of the Diabetes Research & Wellness Foundation, the NHS, the NIHR or the Department of Health.
2) AIRWAVE study
   "Airwave Health Monitoring Study. The Airwave Health Monitoring Study received ethical approval from the National Health Service multi-site research ethics committee (MREC/13/NW/0588). Written informed consent was obtained from all participants."
3) GRAPHIC study
   Genetic Regulation of Arterial Pressure of Humans in the Community (GRAPHIC). The Leicestershire Research Ethics Committee approved the study (6463), and all subjects provided written informed consent.
4) LOLIPOP study
   London Life Sciences Prospective Population (LOLIPOP). The LOLIPOP study is approved by the National Research Ethics Service (07/H0712/150) and all participants gave written informed consent at enrolment.
5) BSMS study
   The Breast Screening and Monitoring Study (BSMS). The London - Roiverside Research Ethics Commitee approved the study (12/LO/2019).

Selected serum/plasma metabolites (n = 22)

SMolESY-select **currently** provides the assignment and relative quantification of 22 serum/plasma metabolites. The present metabolites panel is reported in Table S1 (see below). It is noted that the application of a highly validated commercial algorithm (Bruker IVDr),[4] which employs both 1D and 2D J-RES spectra for the automated assignment/quantification of 40 plasma/serum metabolites, on a random cohort of 2000 plasma spectra, showed that only ~25 metabolites are above limit of detection, appearing in more than 90% of the spectra, whereas the rest (i.e. usually in low abundance) are reported in less than 20% of the spectra. Our metabolites panel encompasses the majority of the most abundant and highly occurrent metabolites in plasma/serum samples and coincides with the 25 metabolites of the above-mentioned commercial software. In addition, all metabolites included in SMolESY-select have been reported as important biomarkers by NMR for a plethora of pathological conditions, nutritional habits, metabolic disorders etc. and a small portion of them is summarised in Table S1. Consequently, the chosen metabolites are of highly importance for any clinical, dietary and metabolomic study.

Table S1. The current panel of metabolites provided by SMolESY-select software – examples of their clinical-metabolic importance.

Serum/plasma metabolites	*Reported as biomarker by NMR*
3-hydroxybutyric acid	• Down-syndrome prediction[5]<br>• Diabetic ketoacidosis[6]<br>• Obesity in females[7]<br>• Human sepsis[8]<br>• Colorectal,[9] pancreatic[10] cancer
Acetic acid	• Mediterranean diet[11]<br>• Alcohol use disorders[12]<br>• Hyperuricemia[13]<br>• Gout[13]<br>• Alzheimer's disease[14]<br>• Metastatic breast cancer[15]
Acetone	• Hyperuricemia[13]<br>• Gout[13]<br>• Coronary heart disease[16]
Alanine	• Prostate cancer[17]<br>• Non-alcoholic steatohepatitis in obese humans[18]
Choline	• Alzheimer's disease[14]<br>• Liver Fibrosis in HCV Patients[19]
Citric acid	• Mediterranean diet[11]<br>• Hyperuricemia[13]<br>• Gout[13]
Creatine	• Pancreatic ductal adenocarcinoma[20]<br>• Metastatic breast cancer[15]
Creatinine	• Hyperuricemia[13]<br>• Gout[13]<br>• Metastatic breast cancer[15]
Dimethyl sulfone	• IBD[21]<br>• Thyroid diseases[22]
Ethanol	• Alcohol use disorders[23]
Formic acid	• Chronic kidneys disease[24]
Glucose	• Hyperuricemia[13]<br>• Gout[13]<br>• Diabetes[25]<br>• Pancreatic ductal adenocarcinoma[20]
Glutamine	• Hyperuricemia[13]<br>• Gout[13]

	• Coronary heart disease[16]
Glycine	• Pancreatic ductal adenocarcinoma[20]<br>• Prostate cancer[17]
Histidine	• Metastatic breast cancer[15]<br>• Coronary heart disease[16]<br>• Non-alcoholic steatohepatitis in obese humans[18]
Isoleucine	• Alzheimer's disease[14]<br>• Pancreatic ductal adenocarcinoma[20]<br>• Coronary heart disease[16]
Lactic acid	• Hyperuricemia[13]<br>• Gout[13]<br>• Pancreatic ductal adenocarcinoma[20]
Leucine	• Hyperuricemia[13]<br>• Gout[13]<br>• Alzheimer's disease[14]<br>• Pancreatic ductal adenocarcinoma[20]<br>• Coronary heart disease[16]<br>• Non-alcoholic steatohepatitis in obese humans[18]
Lysine	• Hyperuricemia[13]<br>• Gout[13]<br>• Coronary heart disease[16]
Phenylalanine	• Hyperuricemia[13]<br>• Gout[13]<br>• Pancreatic ductal adenocarcinoma[20]<br>• Metastatic breast cancer[15]<br>• Non-alcoholic steatohepatitis in obese humans[18]
Tyrosine	• Hyperuricemia[13]<br>• Gout[13]<br>• Pancreatic ductal adenocarcinoma[20]<br>• Non-alcoholic steatohepatitis in obese humans[18]
Valine	• Alzheimer's disease[14]<br>• Pancreatic ductal adenocarcinoma[20]<br>• Coronary heart disease[16]

STOCSY examples for metabolites assignment and signals overlap detection on SMolESY profiles

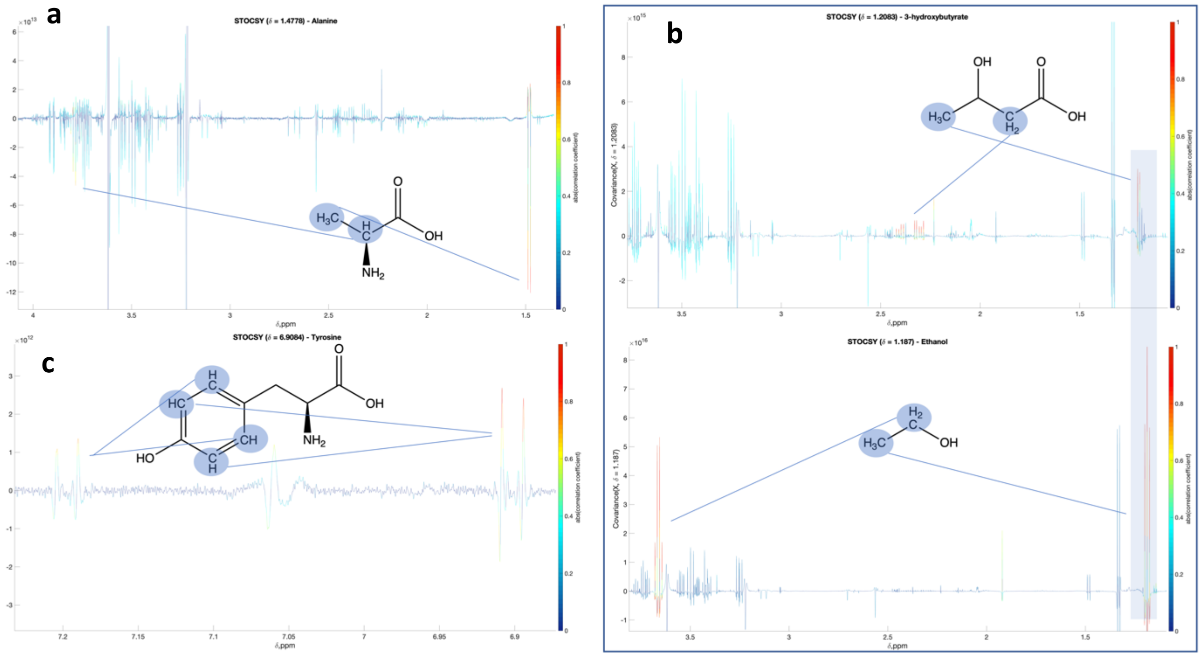

Figure S1. Examples of several metabolites assignment via STOSCY applied on 4023 plasma/serum SMolESY spectra. It is clearly depicted that the application of STOCSY could provide the facile assignment of all ^{1}H NMR spins systems for each metabolite's SMolESY profile, such as for (a) alanine, (b) 3-hydroxybutrate and ethanol and (c) tyrosine. Additionally, STOCSY significantly contributed for the detection of several SMolESY components overlap with other spin systems, as shown in panel (b) for the methyl groups of 3-hydroxybutyrate (i.e. doublet) and ethanol (i.e. triplet).

Further details for automated ^{1}H NMR signals assignment

The chemical shifts of the singlets of glycine, creatinine, creatine, choline and dimethyl sulfone are detected based upon their correlation with the doublet of alanine and/or lactate methyl groups. In detail, 4023 plasma/serum spectra—aligned to glucose anomeric proton— were employed, where the singlets of the above-mentioned metabolites were assigned and correlated with alanine and/or lactate methyl groups NMR signals. The correlations and their combination provided significantly narrow spectral windows (i.e. less than 0.0050 ppm), which combined with the homeostatic nature of blood samples allow the direct detection of the previously mentioned singlets with an infinitesimal probability of mis-assignments. Furthermore, the intra-molecular linear correlations of creatinine's singlets were employed for extra validation of their and creatine's methyl group singlet assignment. The assignment of formic acid, acetone and acetate singlets is performed by their detection (i.e. signals with the maximum intensity) in already predefined spectral windows, whose range was based upon their recorded chemical shifts values in the 4023 unique plasma/serum spectra.

Except for singlets assignment, the linear correlations are also employed for the assignment of ^{1}H NMR signal with various multiplicities (e.g. doublets, triplets etc.) such as the doublet of the 3-hydroxy-butyrate and triplet of ethanol methyl groups as well as the triplet of the -CH_2 group from lysine. Table S2 summarises the correlations between various signals that take place for achieving more robust assignment by employing only one 1D ^{1}H NMR experiment and SMolESY. Finally, the predicted spectral windows based upon correlations for various signals for one plasma spectrum are depicted in Figure S2.

Table S2. The chemical shifts of ^{1}H NMR spins systems from specific metabolites and their correlation on other spin systems chemical shifts.

	Glycine (-CH_2, s, 3.56 ppm)	Creatinine2 (-CH_2, s, 4.05 ppm)	Creatinine1 (-CH_3, s, 3.05 ppm)	Creatine (-CH_3, s, 3.04 ppm)	Choline [-$(CH_3)_3$, s, 3.20 ppm]	DMSO2 [-$(CH_3)_2$, s, 3.15 ppm]	3-hydroxybutyrate (-CH_3, d, 1.20 ppm)	Ethanol (-CH_3, t, 1.18 ppm)	Lysine (-CH_2, t, 3.00 ppm)
Lactate (-CH_3, d, 1.33 ppm)	X	X	X	X		X	X	X	X
Alanine (-CH_3, d, 1.48 ppm)	X				X				
Creatinine2 (-CH_2, s, 4.05 ppm)			X						
Creatinine1 (-CH_3, s, 3.05 ppm)				X					
Creatine (-CH_3, s, 3.04 ppm)			X						

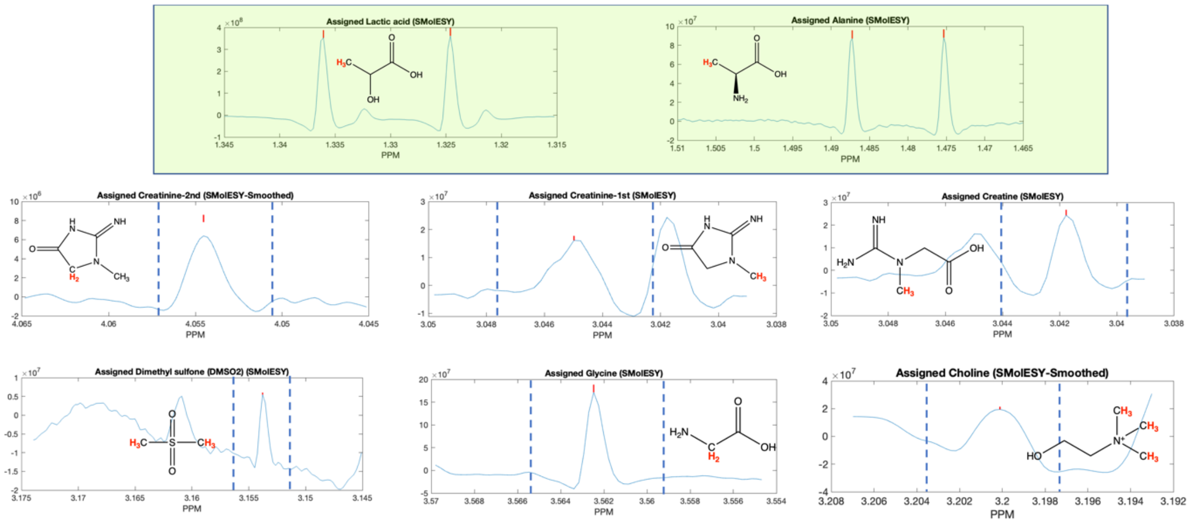

Figure S2. Examples of 5 metabolites singlets assignment based upon their correlation with doublets of lactic acid and alanine methyl groups. The assigned spin systems are in red font, whereas vertical dashed blue lines indicate the "predicted" spectral window for the assignment of each metabolite's corresponding ^{1}H NMR signals. As depicted, the correlation based spectral windows minimize the risk of mis-assignment for singlets.

SMolESY smoothing filter

As previously described,[1] the application of SMolESY to the 1D ^{1}H NMR spectrum depletes broad signals from macromolecules and increases the resolution of small molecule resonances to the expense of decreasing the S/N. This reduction in sensitivity can be easily overcome by the application of a smoothing filter as we previously demonstrated.[1] In detail, the MATLAB (Mathworks) function *smooth* was applied in the selected spectral window, enclosing the spin system of our interest:

Smoothed_signal = smooth(x,y,span), (1)

where, Smoothed_signal are the smoothed data points, x are the ppm datapoints, y are the spectral data and span is the number of data points for calculating the smoothed value (i.e. in our case 11 datapoints were used).
The implementation of SMolESY-select, required the application of smoothing for 5 signals out of 24 included in our software.

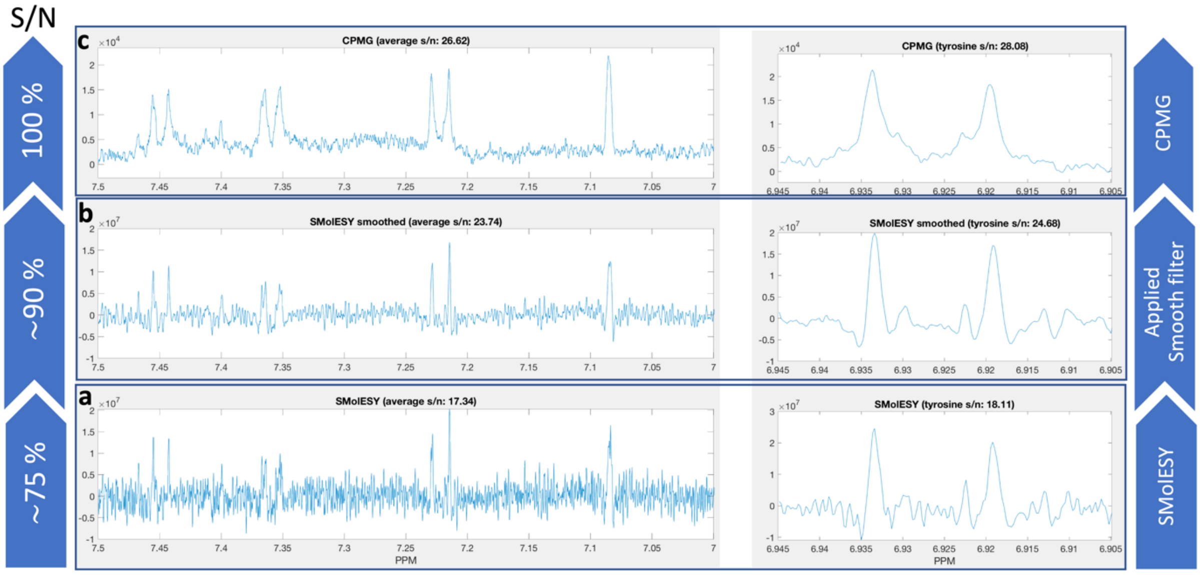

Figure S3. Examples of less populated plasma/serum NMR spectral regions with small molecule sharp NMR signals (i.e. noisy regions). As depicted, applying SMolESY on the ^{1}H NMR spectrum (a) the signal-to-noise ratio (s/n) decreases with respect to the general profile spectrum due to the increase of noise introduced by the NMR data differentiation. (b) The application of a simple and computational fast smoothing filter (moving average) increases this S/N 25 % with respect to SMolESY, resulting in a spectrum only about 10% less sensitive than the corresponding CPMG spectral data. As shown in the panel (b) all filtered SMolESY features are sufficiently denoised facilitating their direct integration, completely deconvolved from the broad ^{1}H NMR signals from lipoproteins, with the clear gain in resolution with respect to CPMG.

Small Molecule Assignment Validation and Spiking Experiments

The optimisation of the defined spectral regions for each spin pattern used in the automatic assignment required the acquisition of a series of 2D $^{1}H,^{1}H$-COSY experiments and spiking experiments using the corresponding standards. SMolESY-select will highlight in red any spectrum where the assignment of the signal of interest has not been possible. Please see below for examples.

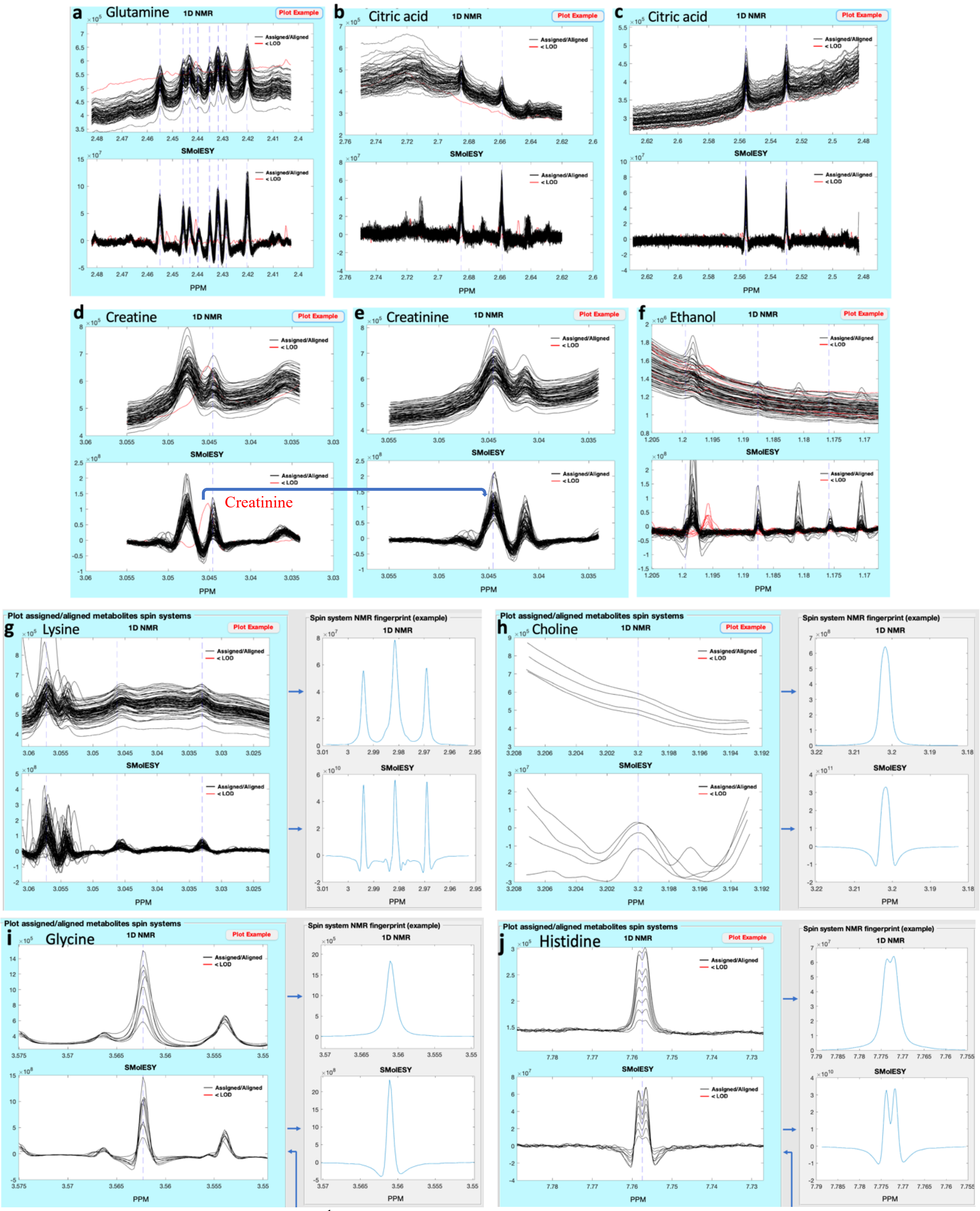

Figure S4. Examples of several assigned ^{1}H NMR spin systems for various metabolites in more than one hundred spectra and assignment validations by spiking experiments, taken as snapshots from the SMolESY-select software (see Figure S9 for more details about our software, available to download here: https://github.com/pantakis/SMolESY-select). All assigned signals for each spin system are aligned in a random position in their predefined spectral window to facilitate the evaluation of the assignment results by user. Here the assigned/aligned signals of specific ^{1}H NMR spin systems from (a) glutamine, (b) citric

acid, (c) creatine, (d) creatinine, (e) ethanol, (g) lysine and (h) choline are depicted. The blue dashed vertical lines indicate all expected components from each NMR fingerprint from the standard 1D ^{1}H NMR experiment and SMolESY. Spectra where the corresponding signal cannot be detected is coloured in red (< limit of detection, LOD, panels a, b and f). Panel (h) presents the automatic assignment of three methyl signals of choline which resonate on the foothills of broad signals from macromolecules. When the metabolite appears in blood in normal ranges, its detection by the standard 1D NMR spectra as well as deconvoluted by fitting algorithms is quite challenging due to signal overlap. However, SMolESY provides an instant deconvolution from the intense baseline and the choline SMolESY signal is easily detected as well as easily integrated. Panel (i) shows the singlet corresponding to the methylene of glycine. Its assignment was confirmed by spiking experiments. SMolESY-select is able to assign this singlet with high confidence as it is completely deconvolved from other nearby signals belonging to different metabolites that are often confused with the glycine signal (e.g. glucerol etc.). Panel (j) depicts the automated assignment of histidine' smoothed SMolESY feature in the same matrix but with various spiked concentrations of histidine.

SMolESY patterns and components for integration

Accurately defined spectral regions are used for the automatic assignment of the different spin-patterns in SMolESY-select. These are defined as SMolESY patterns. The figure below presents the SMolESY patterns used for the assignment of 22 small molecules in blood serum/plasma spectra currently available in SMolESY-select.

Within the SMolESY patterns used for assignment, those components which do not present overlap have been selected for automated integration.

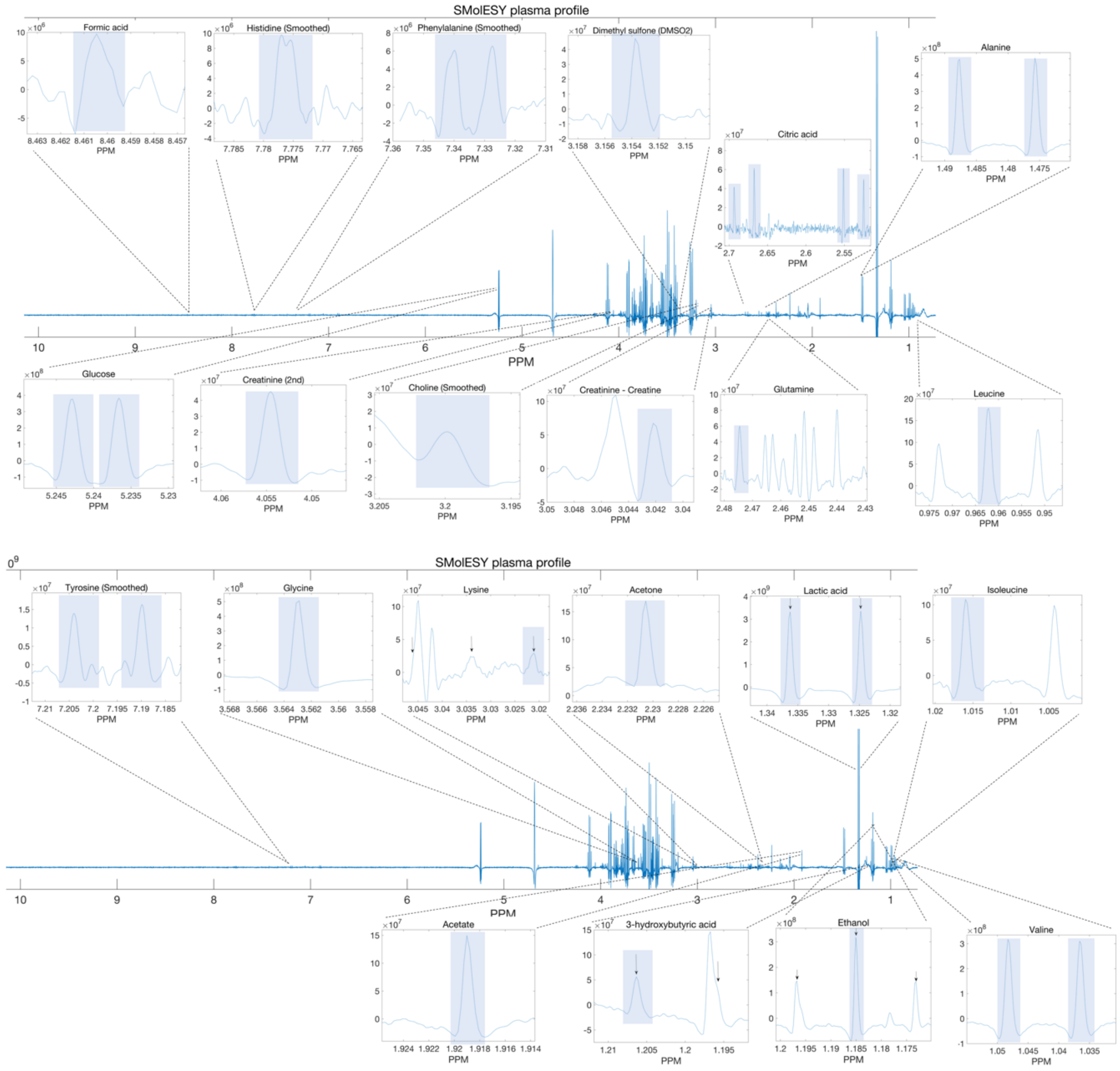

Figure S5. Selected SMolESY components used for integration (i.e. relative quantification) for each ^{1}H NMR spin system SMolESY pattern are highlighted in light blue boxes for each of the 22 assigned metabolites by our algorithm. The selection criteria were based on a thorough inspection of 4023 serum/plasma 2D-JRES spectra and statistical correlation analyses (e.g. STOCSY) among signals and validated on a 8834 plasma/serum cohort.

Validation of SMolESY-select Quantitative Ability: Against experimental Data

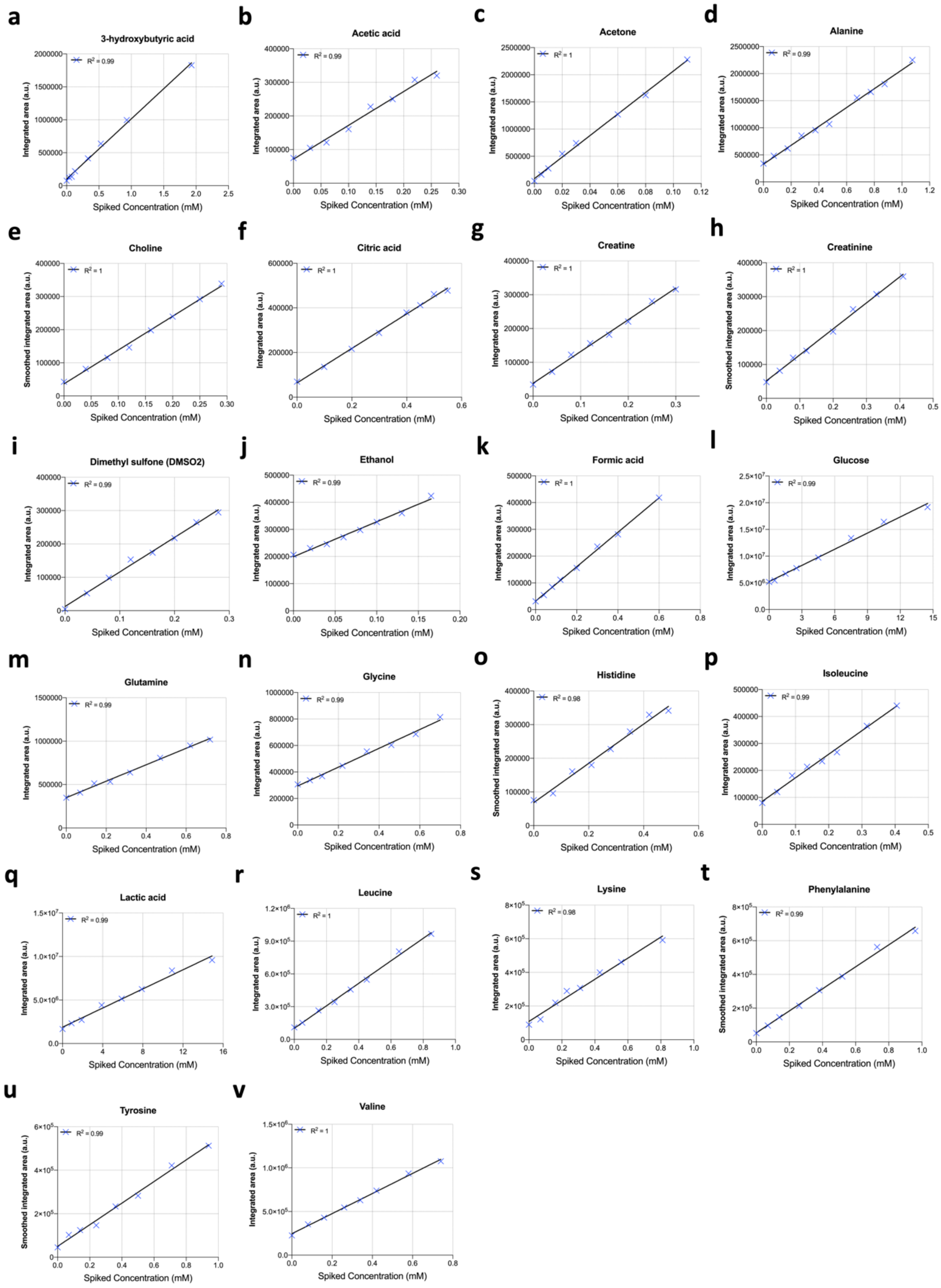

Figure S6. Linear regression analyses between the integrals/proton of the 22 serum/plasma metabolites that are automatically assigned and relatively quantified (integrals are normalised to one proton per metabolite) by our software and seven spiked concentrations of each metabolite into a randomly selected plasma matrix. All SMolESY feature integrals (please see Figure S5 for the SMolESY components that are used for automated integration after their automated assignment by our software) from the 22 metabolites, namely from (a) 3-hydroxybutyric acid, (b) acetic acid, (c) acetone, (d) alanine, (e) choline, (f) citric

acid, (g) creatine, (h) creatinine, (i) DMSO2, (j) ethanol, (k) formic acid, (l) glucose, (m) glutamine, (n) glycine, (o) histidine, (p) isoleucine, (q) lactic acid, (r) leucine, (s) lysine, (t) phenylalanine, (u) tyrosine and (v) valine, exhibit a perfect linear correlation with the spiked amounts of each metabolite, regardless of being smoothed (e.g. for histidine, phenylalanine, choline, creatinine and tyrosine employed SMolESY signals) or not. The spiked concentration values for each metabolite are reported in Table S3. In all linear regression plots, the measured relative concentration on the non-spiked sample is included.

Table S3. The seven spiked concentration values (for the case of alanine nine spiked concentrations were used) for each metabolite in the same randomly selected plasma matrix. To prevent sample degradation and ensure spectrum consistency, each spiked concentration was added to a freshly prepared sample.

Metabolites	Spiked concentrations (mM)							
3-hydroxybutyric acid	0.040	0.080	0.130	0.330	0.530	0.930	1.930	-	-
Acetic acid	0.030	0.060	0.100	0.140	0.180	0.220	0.260	-	-
Acetone	0.005	0.01	0.02	0.03	0.06	0.08	0.11	-	-
Alanine	0.075	0.175	0.275	0.375	0.475	0.675	0.775	0.875	1.075
Choline	0.040	0.080	0.120	0.160	0.200	0.250	0.290	-	-
Citric acid	0.099	0.199	0.299	0.399	0.449	0.499	0.549	-	-
Creatine	0.040	0.080	0.120	0.160	0.200	0.250	0.300	-	-
Creatinine	0.040	0.080	0.120	0.200	0.260	0.330	0.410	-	-
Dimethyl sulfone	0.040	0.080	0.120	0.160	0.200	0.240	0.280	-	-
Ethanol	0.020	0.040	0.060	0.080	0.100	0.130	0.165	-	-
Formic acid	0.040	0.080	0.120	0.200	0.300	0.400	0.600	-	-
Glucose	0.500	1.500	2.500	4.500	7.500	10.500	14.500	-	-
Glutamine	0.070	0.140	0.220	0.320	0.470	0.620	0.720	-	-
Glycine	0.060	0.120	0.220	0.340	0.460	0.580	0.700	-	-
Histidine	0.070	0.140	0.210	0.280	0.350	0.420	0.490	-	-
Isoleucine	0.045	0.09	0.135	0.18	0.225	0.315	0.405	-	-
Lactic acid	0.900	1.900	3.900	5.900	7.900	10.900	14.900	-	-
Leucine	0.050	0.150	0.250	0.350	0.450	0.650	0.850	-	-
Lysine	0.070	0.160	0.230	0.310	0.430	0.560	0.810	-	-
Phenylalanine	0.070	0.140	0.260	0.380	0.520	0.730	0.960	-	-
Tyrosine	0.070	0.140	0.240	0.360	0.500	0.710	0.940	-	-
Valine	0.080	0.160	0.260	0.340	0.420	0.580	0.740	-	-

Validation of SMolESY-select quantitative ability vs. automated quantification by commercial software and clinical measurements

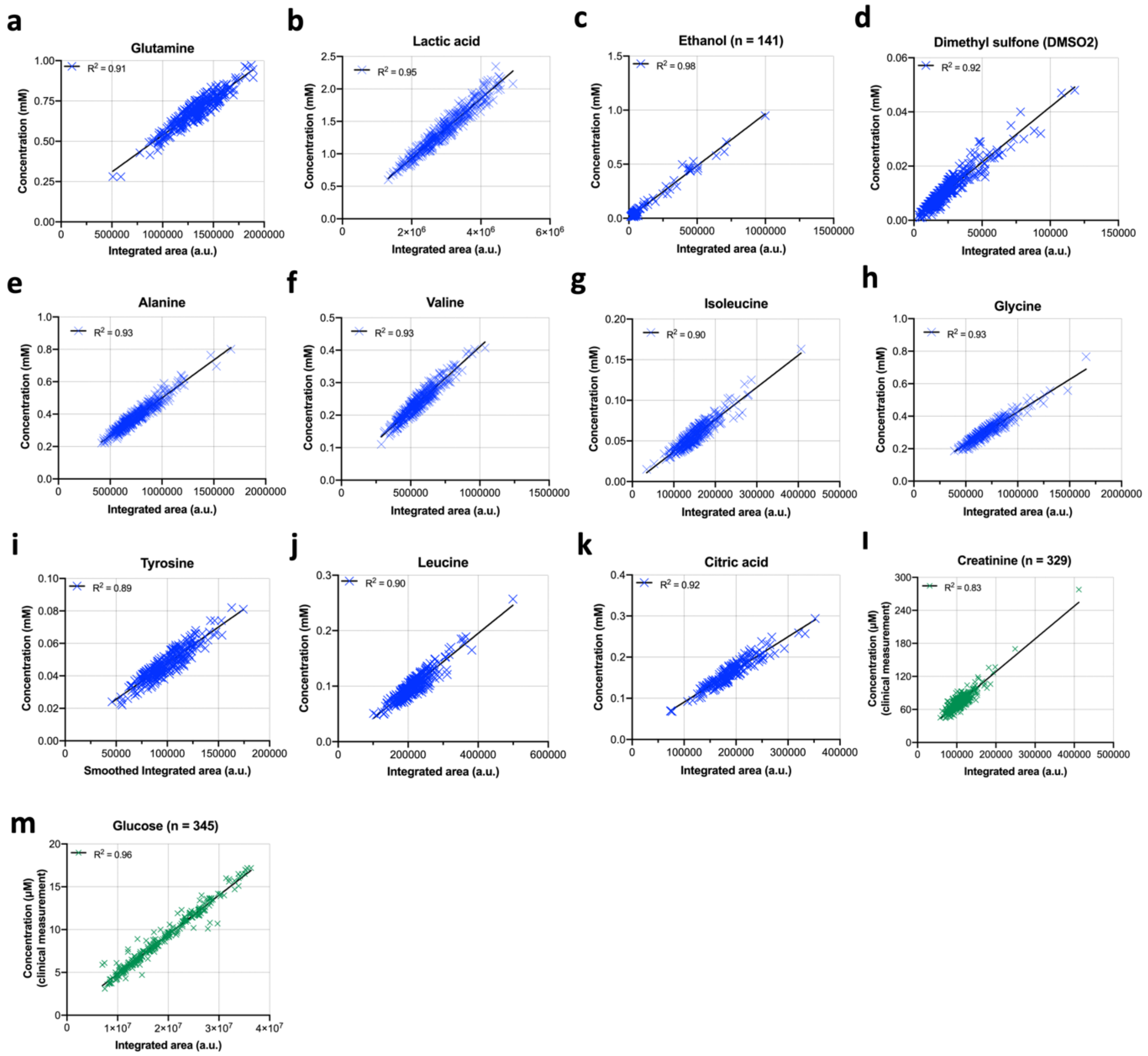

Figure S7. Linear regression analyses of calculated integrals of SMolESY features (normalised to one proton) by our software versus the absolute concentration values calculated by commercial software via fitting/deconvolution algorithms (blue crosses) for (a) glutamine, (b) lactic acid, (c) ethanol (found in 141 out of the 380 plasma samples), (d) dimethyl-sulfone (DMSO2), (e) alanine, (f) valine, (g) isoleucine, (h) glycine, (i) tyrosine, (j) leucine and (k) citric acid and by biochemical (clinical) methods (green crosses) for (l) creatinine and (m) glucose in ~380 plasma samples from a diabetic cohort . In detail, it is clearly observed that our results linearly correlate with those provided by the commercially available BI-Quant-PS platform (Bruker IVDr) as well as clinical methods. Our approach requires a simple integration of one single feature whereas others employ deconvolution approaches that require significantly higher computational investment or laboratory measurements. For instance, the assignment/integration process of 22 metabolites SMolESY features for the 380 plasma spectra required ~1.5 hours, whereas a fitting approach for metabolites signal deconvolution and baseline removal would need an average time of ~6-8 hours on a conventional laptop. This is a substantial discount of computational cost, since our approach could allow the computational targeted analysis of 5k samples per day, whereas the commonly employed computational approaches would require 4 days for the same cohort analysis (note: on a single core calculations).

Combination of targeted/untargeted multivariate analysis on NMR datasets

Two independent NMR plasma-EDTA datasets—consisted of 186 and 175 samples, respectively—were employed to perform untargeted (i.e. using the whole ^{1}H NMR profiles) and targeted (i.e. using the 22 metabolites relative concentrations as automatically measured by SMolESY-select) Principal Component Analysis (PCA). For the untargeted dataset NMR spectra were aligned to glucose anomeric proton at 5.26 ppm, followed by binning (0.02 ppm bin width). PCA results are presented in Figure S8, where both untargeted (left panels) and targeted (left panels) data show the discrimination of the two plasma datasets. Further examination of the loadings plot from the untargeted PCA analysis pointed at glucose and lactate signals, as the main significantly different variables (i.e. metabolites) for the two plasma cohorts. PCA loadings from the targeted dataset showed similar results in this case, which validates both SMolESY-select quantification capability and the value of the targeted datasets, easing the interpretation of metabolomics data as well as expediting any potential biomarkers detection, provided that they belong to the panel of the measured metabolites. While the untargeted approach could lead to the identification of changes in less abundant metabolites, SMolESY-select immediately highlights the most abundant metabolites relevant changes. The analysis below reinforces the validity of our tool and provides an immediate answer of the most abundant metabolites concentration changes.

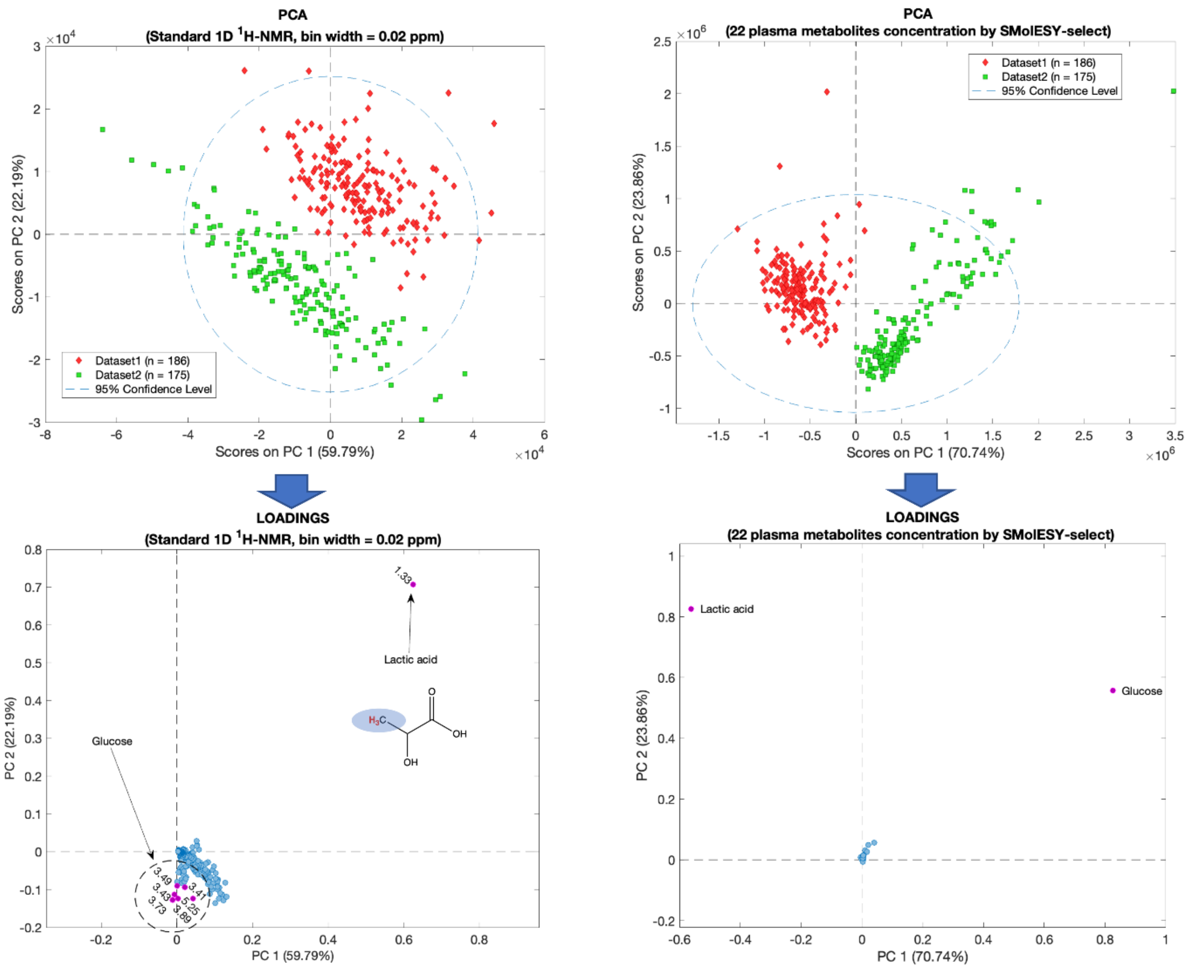

Figure S8. Scores and loadings plot from the PCA analysis of a plasma cohort consisted of two independent NMR plasma-EDTA datasets. The right panels correspond to the untargeted (i.e. the whole bucketed ^{1}H NMR profiles) PCA analysis, whereas the left panels describe the targeted (i.e. 22 plasma metabolites quantification by SMolESY-select for all spectra) PCA results. In the loading plots, the important variables leading to the two datasets discrimination are labelled, highlighting their coincidence.

Overview of the SMolESY-select graphical user interface (GUI)

As depicted below, SMolESY-select is implemented in a totally automated graphical user interface (compiled for both Windows and macOS platforms) that could be freely downloaded at https://github.com/pantakis/SMolESY-select and easily installed. Additionally, our software exhibits several educational features and it could be operated by any user without requiring deep knowledge on NMR spectroscopy. In each step, the user is informed by all running operations and he/she is constantly provided by several interactive plots of the assigned spin systems and the chemical structures of each implemented metabolite so far in our platform.

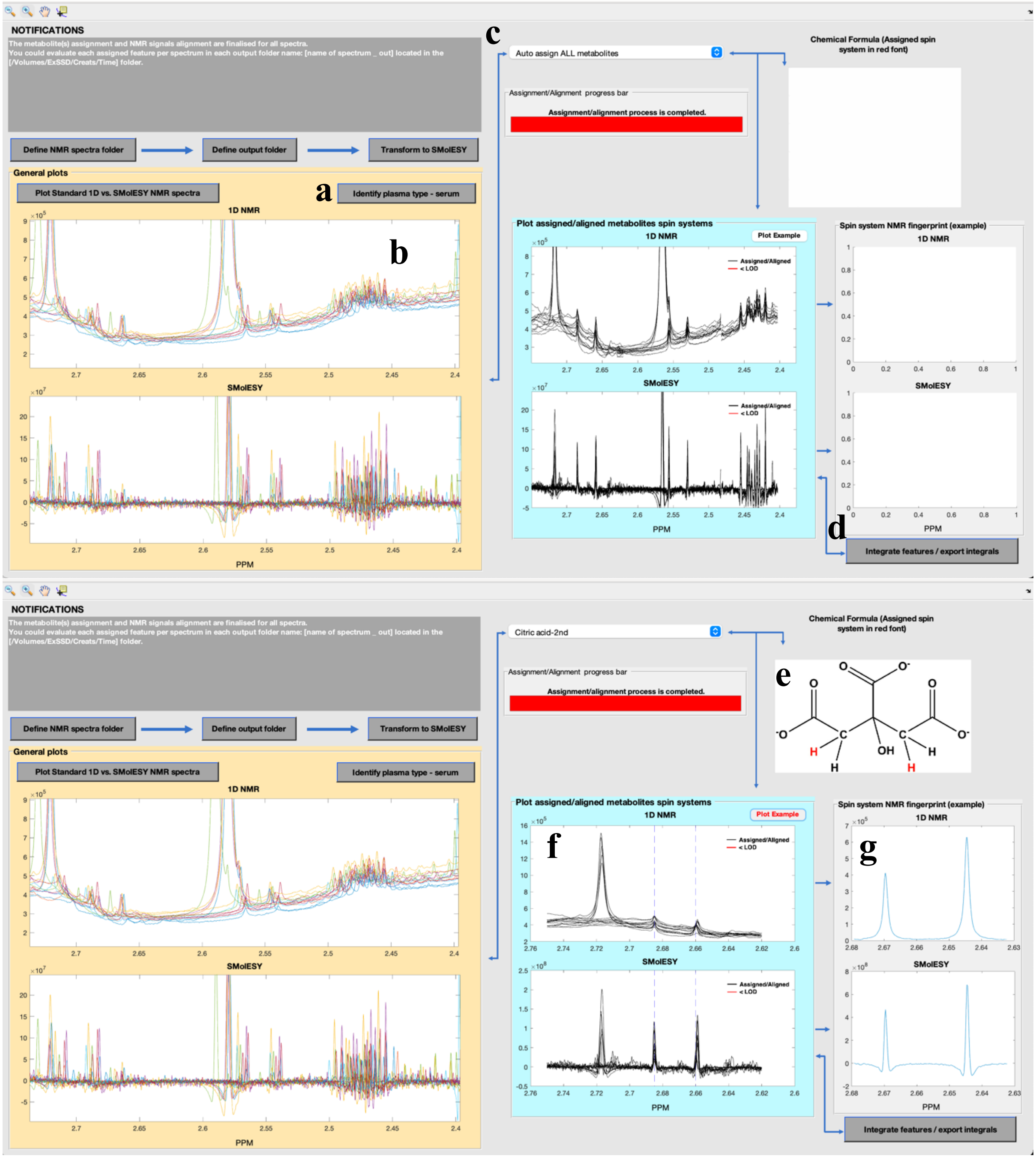

Figure S9. Overview of SMolESY-select graphical user interface (GUI). For the automated assignment/relative quantification, the user needs only to load the standard 1D ^{1}H NMR spectra, define the output folder and transform them to SMolESY (a) One extra function of the software is the automated identification of the blood matrix used for the analysis. Namely, the software automatically detects and informs the user which samples correspond to plasma-EDTA, plasma-Citrate or plasma-Heparin/serum. (b) In addition, the user should plot both SMolESY and 1D NMR spectra for any exploration of the NMR

profiles content. (c) The user could choose from the list the automated assignment of all metabolites at once for all spectra or focus on specific spin systems for each metabolite and (d) afterwards automatically integrate/export integrals of the SMolESY signals for the selected metabolite(s). (e) SMolESY-select informs the user which spin system(s) of each metabolite is selected providing the metabolite's chemical structure and highlighting (by red font) the protons that each assigned SMolESY feature corresponds to. Finally, after the finalised automated assignment, the user could (f),(g) plot as an example the SMolESY/1D ^{1}H NMR signals pattern for each spin system and automatically see (vertical dashed blue lines) the aligned components of each SMolESY/1D ^{1}H NMR pattern.

References

(1) Takis, P. G.; Jiménez, B.; Sands, C. J.; Chekmeneva, E.; Lewis, M. R. SMolESY: An Efficient and Quantitative Alternative to on-Instrument Macromolecular 1H-NMR Signal Suppression. *Chem. Sci.* **2020**, *11* (23), 6000–6011.

(2) Dona, A. C.; Jiménez, B.; Schäfer, H.; Humpfer, E.; Spraul, M.; Lewis, M. R.; Pearce, J. T. M.; Holmes, E.; Lindon, J. C.; Nicholson, J. K. Precision High-Throughput Proton NMR Spectroscopy of Human Urine, Serum, and Plasma for Large-Scale Metabolic Phenotyping. *Anal. Chem.* **2014**, *86* (19), 9887–9894.

(3) Sands, C. J.; Wolfer, A. M.; Correia, G. D. S. G. D. S.; Sadawi, N.; Ahmed, A.; Jimé Nez, B.; Lewis, M. R.; Glen, R. C.; Nicholson, J. K.; Pearce, J. T. M. M.; Jiménez, B.; Lewis, M. R.; Glen, R. C.; Nicholson, J. K.; Pearce, J. T. M. M. The NPYc-Toolbox, a Python Module for the Pre-Processing, Quality-Control and Analysis of Metabolic Profiling Datasets. *Bioinformatics* **2019**, *35* (24), 5359–5360.

(4) Jiménez, B.; Holmes, E.; Heude, C.; Tolson, R. F.; Harvey, N.; Lodge, S. L.; Chetwynd, A. J.; Cannet, C.; Fang, F.; Pearce, J. T. M.; Lewis, M. R.; Viant, M. R.; Lindon, J. C.; Spraul, M.; Schäfer, H.; Nicholson, J. K. Quantitative Lipoprotein Subclass and Low Molecular Weight Metabolite Analysis in Human Serum and Plasma by 1H NMR Spectroscopy in a Multilaboratory Trial. *Anal. Chem.* **2018**, *90* (20), 11962–11971.

(5) Bahado-Singh, R. O.; Akolekar, R.; Mandal, R.; Dong, E.; Xia, J.; Kruger, M.; Wishart, D. S.; Nicolaides, K. Metabolomic Analysis for First-Trimester Down Syndrome Prediction. *Am. J. Obstet. Gynecol.* **2013**, *208* (5), 371.e1-371.e8.

(6) Sheikh-Ali, M.; Karon, B. S.; Basu, A.; Kudva, Y. C.; Muller, L. A.; Xu, J.; Schwenk, W. F.; Miles, J. M. Can Serum β-Hydroxybutyrate Be Used to Diagnose Diabetic Ketoacidosis? *Diabetes Care* **2008**, *31* (4), 643 LP – 647.

(7) Vice, E.; Privette, J. D.; Hickner, R. C.; Barakat, H. A. Ketone Body Metabolism in Lean and Obese Women. *Metab. - Clin. Exp.* **2005**, *54* (11), 1542–1545.

(8) Jaurila, H.; Koivukangas, V.; Koskela, M.; Gäddnäs, F.; Myllymaa, S.; Kullaa, A.; Salo, T.; Ala-Kokko, T. I. 1H NMR Based Metabolomics in Human Sepsis and Healthy Serum. *Metabolites* **2020**, *10* (2), 70.

(9) Ludwig, C.; Ward, D. G.; Martin, A.; Viant, M. R.; Ismail, T.; Johnson, P. J.; Wakelam, M. J. O.; Günther, U. L. Fast Targeted Multidimensional NMR Metabolomics of Colorectal Cancer. *Magn. Reson. Chem.* **2009**, *47 Suppl 1*, S68-73.

(10) OuYang, D.; Xu, J.; Huang, H.; Chen, Z. Metabolomic Profiling of Serum from Human Pancreatic Cancer Patients Using 1H NMR Spectroscopy and Principal Component Analysis. *Appl. Biochem. Biotechnol.* **2011**, *165* (1), 148–154.

(11) Macias, S.; Kirma, J.; Yilmaz, A.; Moore, S. E.; McKinley, M. C.; McKeown, P. P.; Woodside, J. V.; Graham, S. F.; Green, B. D. Application of 1H-NMR Metabolomics for the Discovery of Blood Plasma Biomarkers of a Mediterranean Diet. *Metabolites* **2019**, *9* (10), 201.

(12) Mostafa, H.; Amin, A. M.; Teh, C.-H.; Murugaiyah, V. a/l; Arif, N. H.; Ibrahim, B. Plasma Metabolic Biomarkers for Discriminating Individuals with Alcohol Use Disorders from Social Drinkers and Alcohol-Naive Subjects. *J. Subst. Abuse Treat.* **2017**, *77*, 1–5.

(13) Zhang, Y.; Zhang, H.; Chang, D.; Guo, F.; Pan, H.; Yang, Y. Metabolomics Approach by 1H NMR Spectroscopy of Serum Reveals Progression Axes for Asymptomatic Hyperuricemia and Gout. *Arthritis Res. Ther.* **2018**, *20* (1), 111.

(14) Di Costanzo, A.; Paris, D.; Melck, D.; Angiolillo, A.; Corso, G.; Maniscalco, M.; Motta, A.

Blood Biomarkers Indicate That the Preclinical Stages of Alzheimer's Disease Present Overlapping Molecular Features. *Sci. Rep.* **2020**, *10* (1), 15612.

(15) Jobard, E.; Pontoizeau, C.; Blaise, B. J.; Bachelot, T.; Elena-Herrmann, B.; Trédan, O. A Serum Nuclear Magnetic Resonance-Based Metabolomic Signature of Advanced Metastatic Human Breast Cancer. *Cancer Lett.* **2014**, *343* (1), 33–41.

(16) Song, Z.; Wang, H.; Yin, X.; Deng, P.; Jiang, W. Application of NMR Metabolomics to Search for Human Disease Biomarkers in Blood. *Clin. Chem. Lab. Med. 57* (4), 417–441.

(17) Kumar, D.; Gupta, A.; Mandhani, A.; Sankhwar, S. N. Metabolomics-Derived Prostate Cancer Biomarkers: Fact or Fiction? *J. Proteome Res.* **2015**, *14* (3), 1455–1464.

(18) Männistö, V. T.; Simonen, M.; Hyysalo, J.; Soininen, P.; Kangas, A. J.; Kaminska, D.; Matte, A. K.; Venesmaa, S.; Käkelä, P.; Kärjä, V.; Arola, J.; Gylling, H.; Cederberg, H.; Kuusisto, J.; Laakso, M.; Yki-Järvinen, H.; Ala-Korpela, M.; Pihlajamäki, J. Ketone Body Production Is Differentially Altered in Steatosis and Non-Alcoholic Steatohepatitis in Obese Humans. *Liver Int.* **2015**, *35* (7), 1853–1861.

(19) Embade, N.; Mariño, Z.; Diercks, T.; Cano, A.; Lens, S.; Cabrera, D.; Navasa, M.; Falcón-Pérez, J. M.; Caballería, J.; Castro, A.; Bosch, J.; Mato, J. M.; Millet, O. Metabolic Characterization of Advanced Liver Fibrosis in HCV Patients as Studied by Serum 1H-NMR Spectroscopy. *PLoS One* **2016**, *11* (5), e0155094.

(20) Wen, S.; Zhan, B.; Feng, J.; Hu, W.; Lin, X.; Bai, J.; Huang, H. Non-Invasively Predicting Differentiation of Pancreatic Cancer through Comparative Serum Metabonomic Profiling. *BMC Cancer* **2017**, *17* (1), 708.

(21) Dawiskiba, T.; Deja, S.; Mulak, A.; Zabek, A.; Jawień, E.; Pawełka, D.; Banasik, M.; Mastalerz-Migas, A.; Balcerzak, W.; Kaliszewski, K.; Skóra, J.; Barć, P.; Korta, K.; Pormańczuk, K.; Szyber, P.; Litarski, A.; Młynarz, P. Serum and Urine Metabolomic Fngerprinting in Diagnostics of Inflammatory Bowel Diseases. *World J. Gastroenterol.* **2014**, *20* (1), 163–174.

(22) Wojtowicz, W.; Zabek, A.; Deja, S.; Dawiskiba, T.; Pawelka, D.; Glod, M.; Balcerzak, W.; Mlynarz, P. Serum and Urine 1H NMR-Based Metabolomics in the Diagnosis of Selected Thyroid Diseases. *Sci. Rep.* **2017**, *7* (1), 9108.

(23) Ibrahim, B.; Gopalsamy, K. Chapter 59 - Metabolomics to Differentiate Alcohol Use Disorders From Social Drinkers and Alcohol-Naive Subjects; Preedy, V. R. B. T.-N. of A., Ed.; Academic Press, 2019; pp 577–584.

(24) Davies, R. The Metabolomic Quest for a Biomarker in Chronic Kidney Disease. *Clin. Kidney J.* **2018**, *11* (5), 694–703.

(25) Lin, H.-T.; Cheng, M.-L.; Lo, C.-J.; Lin, G.; Lin, S.-F.; Yeh, J.-T.; Ho, H.-Y.; Lin, J.-R.; Liu, F.-C. 1H Nuclear Magnetic Resonance (NMR)-Based Cerebrospinal Fluid and Plasma Metabolomic Analysis in Type 2 Diabetic Patients and Risk Prediction for Diabetic Microangiopathy. *J. Clin. Med.* **2019**, *8* (6), 874.
